# Synthesis of Inorganic Compounds in the Matrix of Polysaccharide Chitosan

**DOI:** 10.3390/biomimetics6030045

**Published:** 2021-07-05

**Authors:** Larisa Zemskova, Vladimir Silant’ev, Eduard Tokar, Andrei Egorin

**Affiliations:** Institute of Chemistry, Far Eastern Branch, Russian Academy of Sciences, Prospect 100-letiya Vladivostok, 159, 690022 Vladivostok, Russia; zemskova@ich.dvo.ru (L.Z.); vladimir.silantyev@gmail.com (V.S.); d.edd@mail.ru (E.T.)

**Keywords:** chitosan, nanocomposites, metal oxides, metal hydroxides, ferrocyanide, hydroxyapatite, fabrication method

## Abstract

Data related to the fabrication of hybrid materials based on the polysaccharide chitosan were systematized and reviewed. The possibility of using chitosan as a “host” matrix for in situ synthesis of inorganic compounds for the preparation of various types of composite materials were investigated. Coprecipitation of metal oxides/hydroxides (Fe, Ni, Al, Zr, Cu and Mn) with chitosan was carried out through the alkalinization of solutions containing metal salts and chitosan, with the addition of ammonia or alkali solutions, homogeneous hydrolysis of urea, or electrophoretic deposition on the cathode. The synthesis of transition metal ferrocyanides and hydroxyapatite was achieved from precursor salts in a chitosan solution with simultaneous alkalinization. The mechanism of composite formation during the coprecipitation process of inorganic compounds with chitosan is discussed. Composite materials are of interest as sorbents, coatings, sensors, and precursors for the production of ceramic and electrode materials.

## 1. Introduction

Recently, there has been increasing interest in hybrid materials in which inorganic components are introduced into a polymer matrix. These materials enable advances in the fields of application of nanodispersed metal powders, metal oxides/hydroxides, and some salts for use as sorbents, catalysts, and sensors in forms acceptable for practical application, such as granules, membranes, films, and fibers [1,2,3], due to the possibility of preserving the dispersity of nanoparticles (NPs) by preventing their agglomeration and oxidation. The use of biopolymers for the immobilization of inorganic nanoparticles constitutes a new trend in the development of chemistry of nanomaterials (especially biodegradable and biocompatible ones) in various fields, particularly in biomedicine. Therefore, these biopolymer (host)/immobilized nanoparticle (guest) systems serve as a convenient medium for the realization of several chemical processes that are often unavailable under the so-called “traditional” performance conditions of chemical reactions in solutions or solid phase. Examples of these systems include gelatin-immobilized ferrocyanides (FOC) of transition metals, comprising nanoreactors for the synthesis of new compounds [4], or the gelatin–calcium phosphate system [5], which can be used for creating ceramic composite materials in bone tissue engineering [5,6,7].

Hybrid materials based on chitosan (CS) have increasingly found applications in a wide variety of fields, such as water treatment [1,8,9,10,11,12,13,14], biomedicine [2,6,15] and pharmacy [16]. Chitosan-based hybrid materials including conducting polymers [8,16], carbon nanotubes [16,17], and metal and metal oxide nanoparticles [8,9,10,11,12,13,14,15] are under intensive development due to the outstanding properties of the individual components and their observed synergetic effects [16,17].

Chitosan-based nanomaterials are characterized by superior qualities compared to the initial chitosan as a result of the introduction of NPs of various compounds, which impart desirable properties such as high specific surface area, stability, conductivity, photoluminescence sorption properties, and improved mechanical strength [17]. 

Nanoparticles of inorganic compounds can be introduced to chitosan, as in synthetic polymers, through dispersion (immobilization) of pre-fabricated NPs into the polymer solution with subsequent polymer solidification. However, significantly higher interest has been devoted towards the synthesis of materials comprising nanoparticles (guests) in a biopolymer matrix simultaneously with precipitation. However, information on the synthesis of compounds directly in the chitosan matrix is rather insufficient.

The objective of the present work was to demonstrate, on the basis of several examples from the available literature and our own work, possibilities for such syntheses using chitosan as a matrix (host) for the immobilization of inorganic compounds (guests).

## 2. Materials and Methods

Chitosan was purchased from JSC “Vostok-Bor” (Russia); the degree of acetylation was 0.25; the viscosity-average molecular weight was 250 kDa. Nickel chloride (NiCl_2_×6H_2_O), potassium hexacyanoferrate trihydrate (K_4_[Fe(CN)_6_]×3H_2_O, calcium chloride (CaCl_2_), dipotassium hydrogen phosphate (K_2_HPO_4_×3H_2_O), urea (CO(NH_2_)_2_), ammonium hydroxide (NH_4_OH), hydrochloric acid (HCl), and sodium hydroxide (NaOH) were purchased from Nevareaktiv, Russia. All chemicals were of analytical grade and were used as received without further purification. Activated carbon fiber (ACF) Aktilen (B brand) with the specific surface area of 700 m^2^/g was purchased from LenNII “Khimvolokno” and used as the initial activated carbon fiber.

Morphology of the composite materials and distribution of the inorganic component in the bulk was investigated using a Lyra3 XMH (Tescan) scanning electron microscope equipped with an AZtecEnergy energy dispersive X-ray (EDX) microanalyzer automated with an X-Max80 detector (Oxford Instruments, Abingdon, UK).

The phase composition and crystallinity of resulting samples were determined by X-ray diffraction analysis (XRD) using a D8 ADVANCE diffractometer with CuKα (λ = 0.15418 nm) incident radiation and SmartLab diffractometer (Rigaku, Japan) with CuKα–radiation in the 2 θ range from 2° to 80° at a scan speed of 0.2°/s. 

Information about some organo-mineral composites, initial reagents, and methods used for their preparation is summarized in Table 1.

## 3. Results

### 3.1. Material Characteristics Ni(OH)_2_/CS and Ni(OH)_2_/CS/ACF

**Figure 1:**
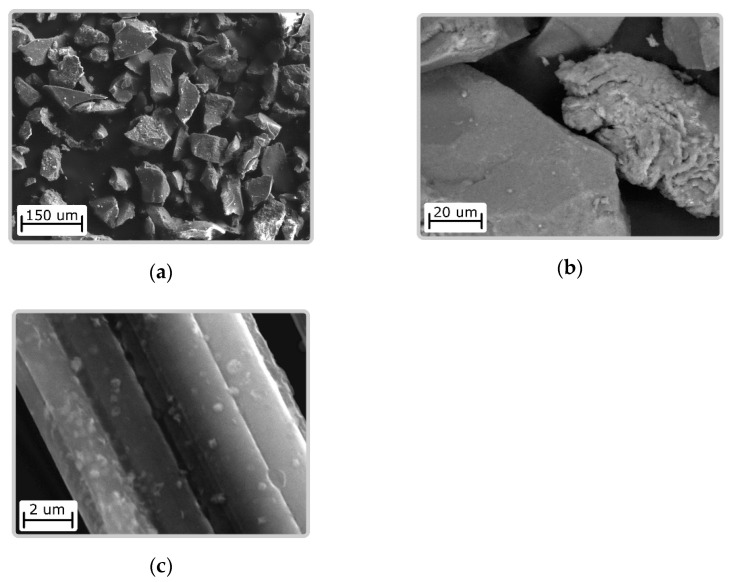
SEM images of samples (**a**,**b**) Ni(OH)_2_/CS with different magnification and (**c**) Ni(OH)_2_/CS/ACF.

Ni(OH)_2_/CS obtained by crushing and subsequent sieving of fractions with a given particle size have an irregular shape. The particles have a dense structure. In the particles deposited on the surface of the carbon fiber, a layered structure is observed (Figure 1).

**Figure 2:**
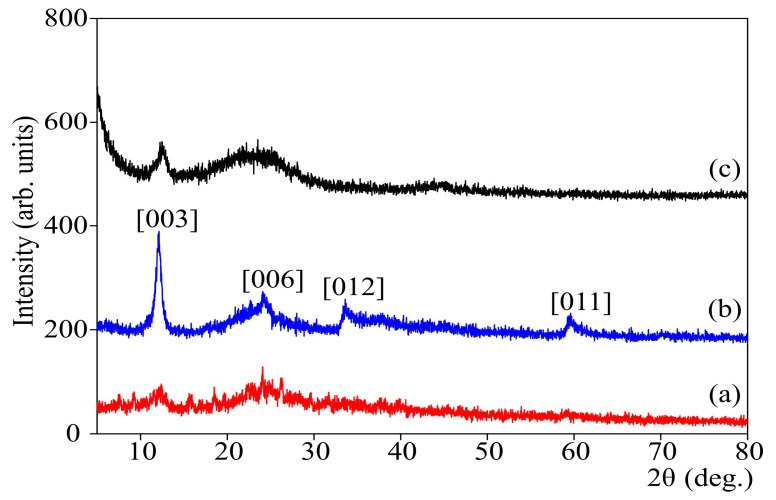
X-ray diffraction patterns of different samples Ni(OH)_2_/CS: (**a**,**b**) were obtained in different experiments; (**c**) was obtained in the presence of ACF as a substrate simultaneously with (**b**).

Figure 2 shows the X-ray diffraction pattern of the “gel-in-gel" synthesis products. Ni(OH)_2_ in the composite sample has a characteristic XRD pattern for α-Ni(OH)_2_ corresponding to a layered turbostratic structure [38].

### 3.2. Characteristics of CFS—Chitosanferrocyanide Sorbent Ni-K, Cu-K, Zn-K

**Figure 3:**
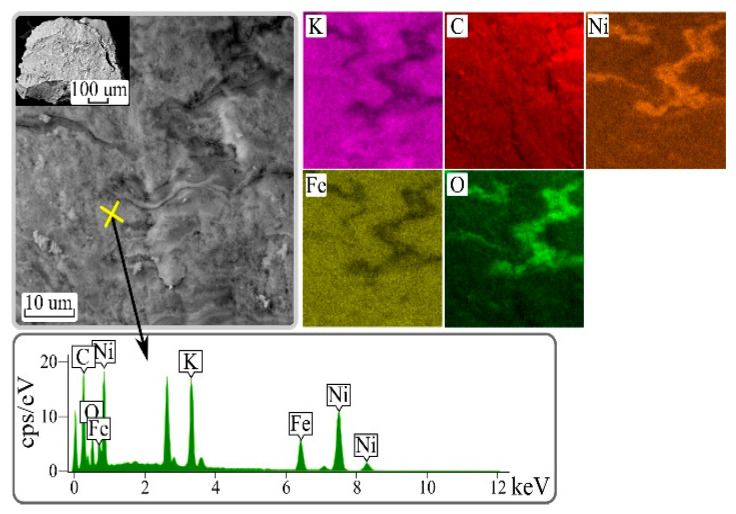
SEM-EDX analysis of the composite CS/FOC K-Ni; X-ray spectrum and distribution of elements K, C, Fe, O, and Ni.

As an example, Figure 3 shows the SEM image of the surface of a particle of the composite sorbent CS/FOC K-Ni. The particles have a dense structure.

**Figure 4:**
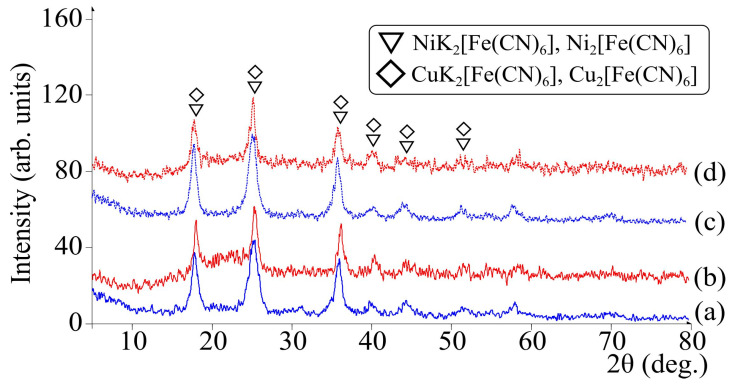
XRD patterns of the samples: initial powders (**a**) FOC K-Ni, (**b**) FOC K-Cu; and chitosan-based hybrids (**c**) CS/FOC K-Ni, (**d**) CS/FOC K-Cu.

The X-ray characteristics of pure FOC powders and hybrid materials, Figure 4, indicate similarities in the compositions of ferrocyanides between powders and composites. The absence of a shift in the position of the reflections on the diffractograms of pure powders and composites indicates insignificant interactions between polymer chitosan and inorganic particles.

### 3.3. Material Characteristics HA/CS

**Figure 5:**
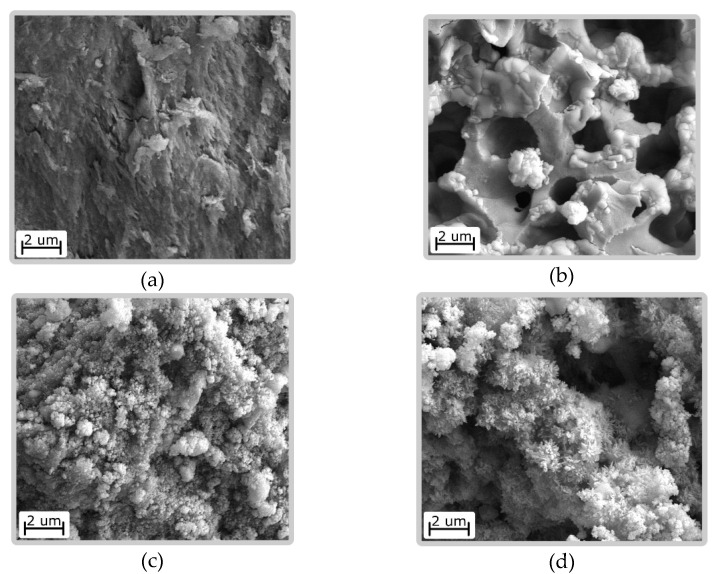
SEM images of samples prepared in atmosphere of NH_3_: (**a**) chitosan film; (**b**) chitosan/phosphate film dried at 100 °C; (**c**) composite produced from chitosan/phosphate film in 0.1 M NaOH during 5 days at 25 °C; (**d**) composite produced from chitosan/phosphate film in 0.1 M NaOH during 12 h at 100 °C.

According to the SEM image data shown in Figure 5 for HA/CS, the sample obtained in the NH_3_ atmosphere has a porous structure.

**Figure 6:**
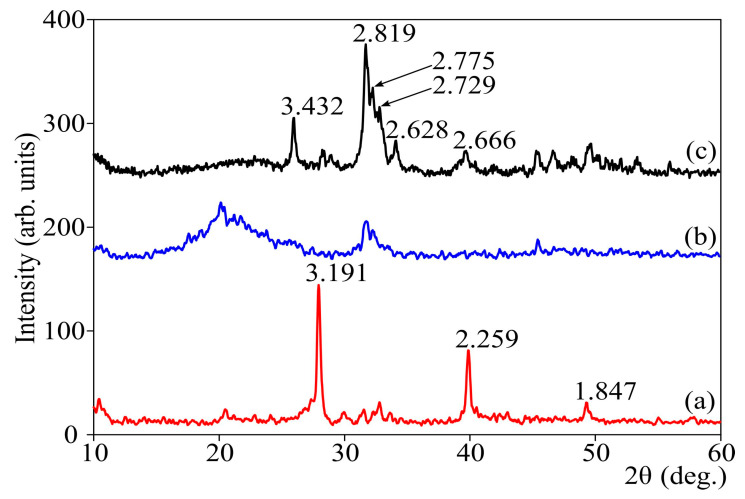
XRD patterns of (**a**) chitosan/phosphate film dried at 100 °C; (**b**) composite produced from chitosan/phosphate film in 0.1 M NaOH during 5 days at 25 °C; (**c**) composite produced from chitosan/phosphate film in 0.1 M NaOH during 12 h at 100 °C. XRD pattern (**c**) corresponds to HA.

Figure 6 shows X-ray patterns of HA/CS samples. In hybrid materials, when the composite is aged in an NaOH solution, HA (b,c) is formed in the polymer volume. With prolonged exposure in a solution of NaOH, CS turns into a crystalline form (b).

## 4. Discussion

Chitosan is a natural polysaccharide produced by N-deacetylation of chitin. As a co-polymer, chitosan is comprised of linear β-(1→4) glycosidiclinkages which are similar in structure to cellulose. In chitosan, 2-acetamido-d-glucose and 2-amino-d-glucose units are combined with glycosidic linkages [17]. Chitosan is a bio-compatible, bio-degradable, bio-renewable, and non-toxic polymer characterized with a variety of useful properties ensuring a substantial interest to hybrid materials on its basis, which can be widely applied as powders, nanoparticles, gel granules, films and membranes, sponges, fibers, or hollow fibers. However, here, the chitosan properties promoting the formation and the fabrication of various organo-inorganic materials is highlighted. The majority of natural polysaccharides, such as pectin, dextrin, agar, agarose, carrageenan, and cellulose, are naturally acidic, whereas chitosan is a highly basic polysaccharide [17].

Chitosan is a natural cationic polysaccharide. It is water-soluble and positively charged and can be obtained by protonation under acidic conditions:

(1) $$CS+H_{3}O^{-}\to CS--H^{+}+H_{2}O$$

Chitosan properties in solutions depend on the molecular weight, degree of deacetylation, pH, and solution ionic strength. At pH 6.5, CS amino groups are deprotonated. As a result of the charge decrease along with the pH increase, the soluble chitosan usually flocculates at pH around 6.0 [39].

Additionally, the polymer could participate in formation of complex compounds consisting of a polymer as a ligand and several transition metal ions bonded to the polymer by a coordination bond [40], which can also form precipitates in alkaline media.

Thus, in alkaline media, chitosan coprecipitation with cations of some metals results in the formation of organo-inorganic composites.

### 4.1. Ni(OH)_2_/Chitosan Nanosize Composites

Among metal hydroxides, nickel hydroxide is most often used in advanced power devices for energy accumulation (batteries and supercapasitors) because of its high theoretical specific capacity, large interlayer distance in the layered structure, excellent pseudo-capacity properties, ease of synthesis, and stability in alkaline electrolytes [41].

Fabrication of nanostructured Ni(OH)_2_ is realized through high-efficiency precipitation methods in the presence of surfactants, and also in the presence of a “green template”—chitosan. The Ni(OH)_2_/CS serves here as an intermediate product in the fabrication of the mesoporous nickel hydroxide [20].

In this study, the fabrication of the Ni(OH)_2_/CS composite is based on the precipitation of nickel hydroxide from aqueous solution of Ni^2+^ during the decomposition of the urea present in the solution. At a temperature of 60 °C, the urea decomposition becomes rather fast, increasing the pH under homogeneous conditions throughout the whole solution volume when precipitation of metal hydroxides becomes possible without creating a high degree of supersaturation. Here, urea acts as a “delay base”, since there is no reaction when it is dissolved in the aqueous metal salt solution at room temperature. The urea hydrolysis starts when temperature increases above 333 K, in accordance with the reaction,

(2) $$CO{\left(NH_{2}\right)}_{2}+3H_{2}O\to CO_{2}+2NH_{4}^{+}+2OH^{-}$$

in which hydroxide ions are released gradually into the homogeneous medium, whereas pH increases throughout the whole solution volume [38]. For nickel hydroxide, the pH of the precipitation start is in the range 6.7 to 7.7, depending on the concentration [42]. In the presence of chitosan in the solution, it is precipitated at pH > 6. Thus, the homogeneous hydrolysis results in the precipitation of the hybrid material Ni(OH)_2_/CS in the presence of chitosan as an external template. Upon the introduction of carbon fiber to the solution as a substrate, the composite is precipitated on the carbon fiber surface.

Figure 2 shows the X-ray diffraction patterns of the products of synthesis of Ni(OH)_2_/CS (“gel in gel”). In the process of synthesis, nickel hydroxide precipitated in the material composition has a characteristic XRD pattern corresponding to α-Ni(OH)_2_ (curve 2) with the turbostratic structure identical to the one reported in [38]. The sizes of Ni(OH)_2_ crystallites contained in the hybrid material are as follows: Ni(OH)_2_/CS, 100 °C—9.48 nm; Ni(OH)_2_/CS/ACF, 100 °C—3.75 nm.

However, it is worth noting that poor reproducibility was observed in our experimental results. In repeated syntheses, compounds are formed preceding α-Ni(OH)_2_, with a maximum diffraction at 2 θ ~12° and 22° and smaller particle sizes (curve 1). The latter could be related to the fact that colloid systems are distinguished by poor property reproducibility.

The surface morphology of the composite materials is shown in Figure 1.

Particles of oxides and hydroxides can be formed using the method of electrosynthesis most appropriate for their growth in a polymer matrix of the polyelectrolyte chitosan with precipitation of compounds or metal–polymer complexes [22,23,24,25].

Metal-containing particles in a polymer matrix were formed as a result of an electrolytic process, in which metal ions or complexes were hydrolyzed by the cathode-generated base with precipitation of hydroxides, oxides, or other insoluble phases, in a manner dependent on the ions present in the electrolyte composition on the substrate surface [43]. The OH^−^-ion-generating cathode reactions are as follows:

(3) $$2H_{2}O+2e^{-}\to H_{2}+2OH^{-}$$

(4) $$O_{2}+2H_{2}O+4e^{-}\to 4OH^{-}$$

Precipitation of nanosize particles on the surface of substrates with highly developed surface and porosity is attractive in terms of using carbon fiber as a cathode. First, the rate of generation of hydroxide ions is rather high. Secondly, the composite material oxide/hydroxide/chitosan is formed as a film on the ACF surface, which is attractive for creating sorbents, electrode materials, and catalysts, in which the processes occur mostly on the material surface, rather than in its bulk.

### 4.2. Transition Metal Ferrocyanides/Chitosan Hybrid Sorbents

Hybrid sorbents based on transition metal ferrocyanides in a chitosan matrix are of interest for cesium removal. Chitosan used as a matrix and insoluble in alkaline media provides a certain stability for ease peptized FOC precipitates, which enables the use of FOC for decontamination in columns and the easy removal of spent sorbents from the reactive medium. The stability of transition metal FOC in the composition of hybrid sorbents applicable for the removal of cesium from high-salinity streams makes it possible to use them in cesium concentration from seawater [44].

There are different methods for the immobilization of FOC into the polysaccharide matrix. In particular, the introduction of FOC into the chitin matrix as granules [45] or foam [46] can be implemented using chitosan. The chitosan solution is added with a prepared FOC suspension, after which granules or discs are formed with subsequent freezing and lyophilic drying and reacetylation of glucosamine groups in order to form chitin.

The synthesis of transition metal ferrocyanides in a chitosan matrix is described in detail in [26,27,28]. In [26], the freshly prepared chitosan resin was saturated with copper(II) and washed, after which wet granules were placed into a solution of K_4_[Fe(CN)_6_] with formation of the resin K_2_Cu_3_[Fe(CN)_6_]_2_ in the phase. However, to obtain a sorbent, granules (spheres) with a water content of 92–96 wt.% were used in order to saturate the resin with copper until a certain capacity. Such resins are prepared in several stages by gelling in appropriate solutions. Grains contain a lot of water, so they are difficult to store and impossible to transport at low temperatures, which limits their practical application. Fabrication of hybrid materials is significantly facilitated by simple mixing of two solutions (acidic chitosan solution containing a transition metal salt and alkaline solution of potassium ferrocyanide) resulting in precipitation of chitosan with simultaneous synthesis of transition metal FOC in the “host” matrix [27,28].

As observed from the XRD data (Figure 4), the use of chitosan had no effect on the structural characteristics of ferrocyanides. The X-ray images correspond to those of pure FOC powders and materials formed ex situ with the introduction of preliminarily obtained particles to the polymer [45,46].

### 4.3. Hybrid Calcium Phosphates/Chitosan Composites

Hydroxyapatite/chitosan (HA/CS) is one of the most often synthesized composites. It can be produced by means of different techniques from the precursors of calcium salts (Ca(NO_3_)_2_, CaCl_2_, Ca(OH)_2_, or CaCO_3_) and orthophosphoric acid salts or orthophosphoric acid. This results in the possibility of synthesizing a complex hydroxyl-/oxyapatite composite consisting of all anions and cations from solution. Different methods have been applied for the fabrication of HA/CS composites: simultaneous coprecipitation with chitosan during ammonia [33,37] or sodium hydroxide [31,35] alkalization, lyophilized composites treatment by sodium hydroxide [32], and electrodeposition on substrate [36].

Hydrolysis of urea-phosphate (NH_2_)_2_CO–H_3_PO_4_ is of great interest because of the slow reagent introduction and solution alkalization [33,34]. Ammonia and sodium hydroxide alkalization are used to increase pH. For example, transformation of brushite into HA is carried out using NaOH. The composite fabrication is usually completed with its lyophilization, which is accompanied by the development of its porous structure [30,31]. 

Introduction of the calcium salt and potassium (ammonium) phosphate to the acidic chitosan solution by the stoichiometric ratio Ca/P = 1.67 results in the formation of hydroxyapatite in accordance with the following reaction [5,15],

(5) $$10Ca{\left(NO_{3}\right)}_{2}+6{\left(NH_{4}\right)}_{2}HPO_{4}+8NH_{3}+2H_{2}O\to Ca_{10}{\left(PO_{4}\right)}_{6}{\left(OH\right)}_{2}+20NH_{4}NO_{3}$$

In the method we developed, alkalization of the mixture of salts and chitosan is realized by holding it, placed in a Petri dish, in an ammonia atmosphere for a long period of time. Thereafter, the precipitate is dried and heated at ~100 °C. Here, hydroxyapatite is not formed, but the formation of crystalline phosphate phase with a non-apatite structure can be observed (Figure 6 curve a), with high content of ammonium chloride (reaction proceeds in HCl from calcium chloride). The transformation of this phase to hydroxyapatite was performed by treatment of the material with a 0.1 M solution of sodium hydroxide at 95–100 °C (or for 72 h at room temperature) (Figure 6, curves b,c) [37].

Phase transformation was corroborated on the basis of the XRD analysis, Figure 6, and SEM data, Figure 5.

## 5. Conclusions

For preparation of composite organo-mineral materials based on chitosan, various methods can be used. These include the deposition and co-deposition of inorganic particles and chitosan in alkaline medium. Additionally, composite films containing oxides and hydroxides in a chitosan matrix can be obtained by cathode electrodeposition based on electrophoretic deposition of chitosan and electrosynthesis of inorganic nanoparticles. 

Ultrathin nanosheets of α-Ni(OH)_2_ in a chitosan matrix were synthesized by using homogeneous hydrolysis of urea at elevated temperature. In the process of increasing pH homogeneously in the bulk solution, the simultaneous formation of nickel hydroxide and chitosan gels occurs. Precipitation and subsequent drying lead to the production of the Ni(OH)_2_/CS composite.

Immobilization of transition metal ferrocyanides into a chitosan matrix to obtain effective sorbents for Cs was carried out by the precipitation method. When mixing two solutions of salts of precursors of FOC—a chitosan solution containing a transition metal salt (Ni, Cu, Zn) and an alkaline ferrocyanide solution—mixed ferrocyanides FOC M-K (M- Ni, Cu, Zn) of the required composition are formed. The composition and content of ferrocyanides in chitosan matrix can be easy controlled by changing the concentration of salts.

Biocomposite materials based on chitosan and calcium phosphates were obtained. Depending on the pH, drying and washing conditions, different phases are formed. The most suitable conditions for synthesis of the phosphate phase of non-apatite structure were homogeneous precipitation using NH_3_ vapors for treatment of chitosan solution containing HA precursors at a molar ratio of Ca/P of 1.67. Hydroxyapatite as the main phosphate phase was detected after treatment of this phase with 0.1 M NaOH.

## Figures and Tables

**Table 1:** Conditions for the formation of hybrid materials based on chitosan.

Composite Material	Application Target Product	Synthesis Conditions	References
Fe_3_O_4_/CS	Sorbent for Sr	Mixture of solutions of Fe (III) and Fe (II) salts (molar ratio 1:2) was added into a 1% CS solution (in 0.1 M HCl), stirred and then NH_4_OH added until neutral reaction, washed, filtered, air-dried, heated at 100 °C, crushed, and sieved.	[18,19]
Fe(OH)_3_/CS	Sorbent for Sr	Similar procedure, solution of Fe(III) salt was added into the CS solution.	[18]
Ni(OH)_2_/CS	Fabrication of nanomaterials	A chitosan solution (2 wt.%) was mixed with nickel nitrate solution at various volumetric ratios of chitosan to nickel nitrate solution of 1:0.5, 1:1, 1:1.5, and 1:2. The chitosan/nickel nitrate mixtures were dripped vertically via a needle into a precipitation bath consisting of 1.5 M NaOH solution using a syringe pump. The dried beads were annealed at 500 and 600 °C.	[20]
Al(OH)_3_/CS Si(OH)_4_/CS	Porous ceramics	The aluminum nitrate aqueous solution was added into chitosan solution under stirring, and then this Al-chitosan solution was added into a NH_4_OH solution (50% v/v) under stirring to form of drops with a syringe. The gel spheres dried at ambient temperature. CS solution mixed with tetraethylorthosilicate (TEOS) and ethanol as a solvent was added into a NH_4_OH solution. The spherical metal oxides (Al and Si) samples were obtained for calcinations of hybrid spheres at 350, 550, and 700 °C.	[21]
Ni(OH)_2_/CS	Electrode material, sorbent	Homogeneous hydrolysis of the NiCl_2_ precursor in the presence of urea CO(NH_2_)_2_ (at a molar ratio of 0.07: 0.5) and CS solution 0.1 wt.% in 0.01 M HCl at 90 ° C for 9 h. The cooled gel was filtered, dried, and heated at 100 °C.	Present study
Ni(OH)_2_/CS/ACF	Electrode material, sorbent	Similar procedure in the presence of ACF as a substrate.	Present study
Al(OH)_3_/CS γ–Fe_2_O_3_ Zr(OH)_4_/CS Ag/CS	Composite films—biomedical implants, antimicrobial coatings, biosensors	Electrodeposition from solutions of ZrO(NO_3_)_2_, Al(NO_3_)_2_, FeCl_3_, and AgNO_3_ water or aqueous-alcoholic solvents containing 0–0.6 g/L CS in galvanostatic mode on Pt or stainless steel foil.	[22]
Ni(OH)_2_/CS/ACF	Electrode material	Electrodeposition from a NiCl_2_ and CS solution in the background electrolyte NaCl in a potentiostatic mode onto an ACF electrode at a potential of −700 (−900) mV related to Ag/AgCl.	[23]
Cu(OH)_2_/CS/ACF	Catalyst, antibacterial coatings	Electrodeposition from a CuCl_2_ and CS solution in the background electrolyte NaCl in a potentiostatic mode onto an ACF electrode at a potential of −700 (−940) mV rel. Ag/AgCl.	[24]
MnO_2_/CS/ACF	Electrode material, sorbent	Electrodeposition from a solution of MnCl_2_ and CS in the background electrolyte NH_4_Cl in a potentiostatic mode onto an ACF electrode at a potential of −700 mV rel. Ag/AgCl with air purging of the electrolyte.	[25]
CFS—chitosan ferrocyanide sorbent K-Cu	Sorbent for Cs	Chitosan granules with a water content of 92–96 wt.% were formed from a solution of chitosan in acetic acid. Then it was saturated with an aqueous solution of Cu(II) sulfate until the copper sorption tank is filled. Then it is treated with a K_4_[Fe(CN)_6_] salt solution.	[26]
CFS—chitosan ferrocyanide sorbent K-Ni, K-Cu, K-Zn (CS/FOC K-Ni, CS/FOC K-Cu, CS/FOC K-Zn)	Sorbent for Cs	The chitosan acidic solution was combined with transition metal salt (Ni, Cu, or Zn), then the obtained mixture was dispersed to the alkaline solution of potassium ferrocyanide. Otherwise (vice versa), the alkaline solution of potassium ferrocyanide was dispersed to the chitosan acidic solution containing a Ni(II) salt. The molar ratio M^2+^/[Fe(CN)_6_]^4−^ = 3:1. The precipitate was filtered and heated at 100 °C.	Present study [27,28]
HA/CS	Composites, films, biomedical coatings, membranes	The solution of Ca(NO_3_)_2_ and CaCl_2_ salts or the suspension of Ca(OH)_2_ and CaCO_3_ in chitosan solution were added with phosphates: (NH_4_)_2_HPO_4_, NaH_2_PO_4_, K_2_HPO_4_, H_3_PO_4_, or urea-phosphate. Then alkalization with NH_4_OH or NaOH. Drying in air or lyophilization.	[29,30,31,32,33,34,35]
HA/CS	Coatings	Electrochemical deposition from the CS solution containing brushite. Conversion of brushite into HA by treatment with alkali 0.1 M NaOH (24 h at 95–100 °C; 72 h at room temperature).	[36]
HA/CS	Films, biomedical coatings, membranes	The CS solution was combined with salts CaCl_2_ and K_2_HPO_4_ at the molar ratio Ca/P = 1.67. The mixture was placed into NH_3_ atmosphere and held there for 1 h until pH ~10. Then the mixture was heated at 100 °C for 12 h. Conversion of the film to by treatment with alkali 0.1 M NaOH (24 h at 95–100 °C; 72 h at room temperature).	Present study [37]

## Data Availability

Not applicable.

## References

[1] Sarkar S., Guibal E., Quignard F., SenGupta A.K. (2012). Polymer-Supported Metals and Metal Oxide Nanoparticles: Synthesis, Characterization, and Applications. J. Nanopart. Res..

[2] Zhang Y., Wu B., Xu H., Liu H., Wang M., He Y., Pan B. (2016). Nanomaterials-Enabled Water and Wastewater Treatment. Nanoimpact.

[3] Šebesta F. (1997). Composite Sorbents of Inorganic Ion-Exchangers and Polyacrylonitrile Binding Matrix. J. Radioanal. Nucl. Chem..

[4] Mikhailov O.V. (2014). Molecular Nanotechnologies of Gelatin-Immobilization Using Macrocyclic Metal Chelates. Nano Rev..

[5] Barinov S.M. (2010). Calcium Phosphate-Based Ceramic and Composite Materials for Medicine. Russ. Chem. Rev..

[6] Dorozhkin S.V. (2009). Nanodimensional and Nanocrystalline Apatites and Other Calcium Orthophosphates in Biomedical Engineering, Biology and Medicine. Materials.

[7] Pighinelli L., Kucharska M. (2013). Chitosan–Hydroxyapatite Composites. Carbohydr. Polym..

[8] Zhang L., Zeng Y., Cheng Z. (2016). Removal of Heavy Metal Ions Using Chitosan and Modified Chitosan: A Review. J. Mol. Liq..

[9] Wang J., Chen C. (2014). Chitosan-Based Biosorbents: Modification and Application for Biosorption of Heavy Metals and Radionuclides. Bioresour. Technol..

[10] Wang X., Liu Y., Zheng J. (2016). Removal of As(III) and As(V) from Water by Chitosan and Chitosan Derivatives: A Review. Environ. Sci. Pollut. Res..

[11] Pontoni L., Fabbricino M. (2012). Use of Chitosan and Chitosan-Derivatives to Remove Arsenic from Aqueous Solutions—A Mini Review. Carbohydr. Res..

[12] Reddy D.H.K., Lee S.-M. (2013). Application of Magnetic Chitosan Composites for the Removal of Toxic Metal and Dyes from Aqueous Solutions. Adv. Colloid Interface Sci..

[13] Gómez-Pastora J., Bringas E., Ortiz I. (2014). Recent Progress and Future Challenges on the Use of High Performance Magnetic Nano-Adsorbents in Environmental Applications. Chem. Eng. J..

[14] Liu S., Huang B., Chai L., Liu Y., Zeng G., Wang X., Zeng W., Shang M., Deng J., Zhou Z. (2017). Enhancement of As( v ) Adsorption from Aqueous Solution by a Magnetic Chitosan/Biochar Composite. RSC Adv..

[15] Boudemagh D., Venturini P., Fleutot S., Cleymand F. (2019). Elaboration of Hydroxyapatite Nanoparticles and Chitosan/Hydroxyapatite Composites: A Present Status. Polym. Bull..

[16] Sunil D. (2013). Recennt Advances on Chitosan-Metal Oxide Nanoparticles and Their Biological Application. Mater. Sci. Forum.

[17] Shukla S.K., Mishra A.K., Arotiba O.A., Mamba B.B. (2013). Chitosan-Based Nanomaterials: A State-of-the-Art Review. Int. J. Biol. Macromol..

[18] Zemskova L., Egorin A., Tokar E., Ivanov V., Bratskaya S. (2018). New Chitosan/Iron Oxide Composites: Fabrication and Application for Removal of Sr2+ Radionuclide from Aqueous Solutions. Biomimetics.

[19] Egorin A., Tokar E., Matskevich A., Ivanov N., Tkachenko I., Sokolnitskaya T., Zemskova L. (2020). Composite Magnetic Sorbents Based on Iron Oxides in Different Polymer Matrices: Comparison and Application for Removal of Strontium. Biomimetics.

[20] Choo C.K., Goh T.L., Shahcheraghi L., Ngoh G.C., Abdullah A.Z., Horri B.A., Salamatinia B. (2016). Synthesis and Characterization of NiO Nano-Spheres by Templating on Chitosan as a Green Precursor. J. Am. Ceram. Soc..

[21] Braga T.P., Gomes E.C.C., de Sousa A.F., Carreño N.L.V., Longhinotti E., Valentini A. (2009). Synthesis of Hybrid Mesoporous Spheres Using the Chitosan as Template. J. Non-Cryst. Solids.

[22] Zhitomirsky I., Hashambhoy A. (2007). Chitosan-Mediated Electrosynthesis of Organic–Inorganic Nanocomposites. J. Mater. Process. Technol..

[23] Zemskova L.A., Nikolenko Y.M., Sheveleva I.V., Voit A.V., Kuryavyi V.G., Sergienko V.I. (2011). Hybrid Nickel Oxide-Carbon Fiber Composites Obtained in the Presence of Surfactants. Glass Phys. Chem..

[24] Zemskova L.A., Voit A.V., Kaidalova T.A., Barinov N.N., Nikolenko Y.M., Ziatdinov A.M. (2012). Organic-Mineral Composites Copper Oxide/Chitosan/Carbon Fiber Obtained by the Electrodeposition Method. Russ. J. Appl. Chem..

[25] Zemskova L.A., Voyt A.V., Barinov N.N., Kaydalova T.A. (2014). Functional Materials Based on Manganese Dioxide Deposited on Carbon Fiber. Glass Phys. Chem..

[26] Rumyantseva E.V., Veleshko A.N., Kulyukhin S.A., Veleshko I.E., Shaitura D.S., Rozanov K.V., Dmitrieva N.A. (2009). Preparation and Properties of Modified Spherically Granulated Chitosan for Sorption of 137Cs from Solutions. Radiochemistry.

[27] Egorin A., Tokar E., Zemskova L. (2016). Chitosan-Ferrocyanide Sorbent for Cs-137 Removal from Mineralized Alkaline Media. Radiochim. Acta.

[28] Zemskova L., Egorin A., Tokar E., Ivanov V. (2019). Chitosan-Based Biosorbents: Immobilization of Metal Hexacyanoferrates and Application for Removal of Cesium Radionuclide from Aqueous Solutions. J. Sol-Gel Sci. Technol..

[29] Yamaguchi I., Tokuchi K., Fukuzaki H., Koyama Y., Takakuda K., Monma H., Tanaka J. (2001). Preparation and Microstructure Analysis of Chitosan/Hydroxyapatite Nanocomposites. J. Biomed. Mater. Res..

[30] Chen J., Nan K., Yin S., Wang Y., Wu T., Zhang Q. (2010). Characterization and Biocompatibility of Nanohybrid Scaffold Prepared via in Situ Crystallization of Hydroxyapatite in Chitosan Matrix. Colloids Surf. B Biointerfaces.

[31] Nikpour M.R., Rabiee S.M., Jahanshahi M. (2012). Synthesis and Characterization of Hydroxyapatite/Chitosan Nanocomposite Materials for Medical Engineering Applications. Compos. Part B Eng..

[32] Kong L., Gao Y., Cao W., Gong Y., Zhao N., Zhang X. (2005). Preparation and Characterization of Nano-Hydroxyapatite/Chitosan Composite Scaffolds. J. Biomed. Mater. Res. Part A.

[33] Rogina A., Ivanković M., Ivanković H. (2013). Preparation and Characterization of Nano-Hydroxyapatite within Chitosan Matrix. Mater. Sci. Eng. C.

[34] Rogina A., Rico P., Gallego Ferrer G., Ivanković M., Ivanković H. (2015). Effect of in Situ Formed Hydroxyapatite on Microstructure of Freeze-Gelled Chitosan-Based Biocomposite Scaffolds. Eur. Polym. J..

[35] Danilchenko S.N. (2009). Chitosan–Hydroxyapatite Composite Biomaterials Made by a One Step Co-Precipitation Method: Preparation, Characterization and in Vivo Tests. JBPC.

[36] Redepenning J., Venkataraman G., Chen J., Stafford N. (2003). Electrochemical Preparation of Chitosan/Hydroxyapatite Composite Coatings on Titanium Substrates. J. Biomed. Mater. Res. Part A.

[37] Silant’ev V.E., Egorkin V.S., Zemskova L.A., Sinebryukhov S.L., Gnedenkov S.V. (2020). Synthesis of Phosphate Phases on Polysaccharide Template. Solid State Phenom..

[38] Mavis B. (2003). Homogeneous Precipitation of Nickel Hydroxide Powders.

[39] Zhitomirsky I. (2006). Electrophoretic Deposition of Organic–Inorganic Nanocomposites. J. Mater. Sci..

[40] Gerente C., Lee V.K.C., Cloirec P.L., McKay G. (2007). Application of Chitosan for the Removal of Metals From Wastewaters by Adsorption—Mechanisms and Models Review. Crit. Rev. Environ. Sci. Technol..

[41] Lokhande P.E., Pawar K., Chavan U.S. (2018). Chemically Deposited Ultrathin α-Ni(OH)_2_ Nanosheet Using Surfactant on Ni Foam for High Performance Supercapacitor Application. Mater. Sci. Energy Technol..

[42] Lurie J. (1975). Handbook of Analytical Chemistry.

[43] Zhitomirsky I. (2002). Cathodic Electrodeposition of Ceramic and Organoceramic Materials. Fundamental Aspects. Adv. Colloid Interface Sci..

[44] Egorin A., Tokar E., Zemskova L., Didenko N., Portnyagin A., Azarova Y., Palamarchuk M., Tananaev I., Avramenko V. (2017). Chitosan-Ferrocyanide Sorbents for Concentrating Cs-137 from Seawater. Sep. Sci. Technol..

[45] Vincent T., Vincent C., Barré Y., Guari Y., Saout G.L., Guibal E. (2014). Immobilization of Metal Hexacyanoferrates in Chitin Beads for Cesium Sorption: Synthesis and Characterization. J. Mater. Chem. A.

[46] Vincent C., Hertz A., Vincent T., Barré Y., Guibal E. (2014). Immobilization of Inorganic Ion-Exchanger into Biopolymer Foams—Application to Cesium Sorption. Chem. Eng. J..

